# The Effectiveness of Social Marketing Interventions to Improve HIV Testing Among Gay, Bisexual and Other Men Who Have Sex with Men: A Systematic Review

**DOI:** 10.1007/s10461-019-02507-7

**Published:** 2019-04-20

**Authors:** Lisa McDaid, Julie Riddell, Gemma Teal, Nicola Boydell, Nicky Coia, Paul Flowers

**Affiliations:** 1 MRC/CSO Social and Public Health Sciences Unit, University of Glasgow, 200 Renfield Street, Glasgow, Scotland G2 3AX UK; 2 Institute of Design Innovation, Glasgow School of Art, Glasgow, Scotland UK; 3 Usher Institute of Population Health Sciences and Informatics, University of Edinburgh, Edinburgh, Scotland UK; 4 NHS Greater Glasgow and Clyde, Glasgow, Scotland UK

**Keywords:** HIV testing, Social Marketing, Men who have sex with men, Systematic review, Mass media, Communications

## Abstract

HIV testing is central to biomedical HIV prevention, but testing among men who have sex with men remains suboptimal. We evaluated effectiveness of mass media and communication interventions to increase HIV testing and explored patterns between study type, internal validity and intervention effectiveness for the first time. Five databases were searched for articles published between 2009 and 2016 using standard MeSH terms. Eligible studies were quality appraised using standard checklists for risk of bias. Data were extracted and synthesised narratively. Nineteen studies met inclusion criteria; 11 were cross-sectional/non-comparative studies, four were pre/post or interrupted time series, three were randomised controlled trials (RCTs) and one was a case study. Risk of bias was high. Five cross-sectional (two graded as high internal validity, one medium and two low) and one RCT (medium validity) reported increased HIV testing. Further work is required to develop and evaluate interventions to increase frequency and maintenance of HIV testing.

**Electronic supplementary material:**

The online version of this article (10.1007/s10461-019-02507-7) contains supplementary material, which is available to authorized users.

## Resumen

Las pruebas del VIH son fundamentales para la prevención biomédica de esa enfermedad, pero la adherencia a esas pruebas entre hombres que tienen relaciones sexuales con hombres sigue siendo subóptima. Hemos evaluado la efectividad de los medios masivos y las intervenciones de comunicación para aumentar la adherencia a las pruebas de VIH. También hemos explorado los patrones entre el tipo de estudio, la validez interna y la efectividad de la intervención por primera vez. Se realizaron búsquedas en cinco bases de datos de artículos publicados entre 2009–2016 utilizando términos estándar MeSH. Los estudios elegibles se evaluaron por calidad utilizando listas de verificación estándar para el riesgo de sesgo. Los datos fueron extraídos y sintetizados narrativamente. Diecinueve estudios cumplieron los criterios de inclusión; once fueron estudios transversales/no comparativos, cuatro fueron series temporales pre/post o interrumpidas, tres fueron ensayos controlados aleatorios (ECA) y uno fue un estudio de caso. El riesgo de sesgo fue alto. Cinco estudios transversales (dos de alta validez interna, uno de media y dos de baja) así como un ECA (de validez media) reportaron un incremento en el número de pruebas de VIH. Se requiere más trabajo para desarrollar y evaluar intervenciones para aumentar la frecuencia y el mantenimiento de las pruebas de VIH.

## Introduction

HIV testing is increasingly central to wider approaches to HIV prevention for both those testing positive (Treatment as prevention, TasP) and those testing negative (Pre-exposure prophylaxis, PrEP). Public Health England estimated that 13% of HIV positive men who have sex with men (MSM) were undiagnosed in 2016 [1], and previous research suggests that delays in HIV diagnosis are associated with poorer health outcomes and treatment response, increased mortality and healthcare cost, and increased levels of onward transmission [2, 3]. Current UK guidelines recommend annual testing for all MSM, with more frequent testing (e.g. three monthly) recommended for men at higher risk of HIV infection [4]. However, recent research based on the findings of community-based surveys found that only half of UK gay, bisexual and other men who have sex with men (GBMSM) reported annual testing, with less than one quarter of GBMSM defined as ‘at risk’ of HIV testing more frequently [5]. HIV testing is associated with age, ethnicity and educational attainment, while multiple factors influence testing decisions, including fear of a positive test result and self-perceived risk [6, 7].

HIV testing is a complex behavioural domain and different types of intervention, which focus on increasing knowledge, awareness, access via varied settings and reducing barriers to testing, have been attempted to increase HIV testing among different population groups [8]. Mass media interventions are one approach that can be used to increase awareness of testing (others include social media, and one-to-one, opportunistic and group-based information provision) and have increasingly been recognized as a powerful tool in sharing public health messages [8–10]. Wei et al. conducted a systematic review exploring the impact of social marketing interventions on HIV/STI testing uptake among MSM and transgender women and found only three studies that met their inclusion criteria. Although these studies seemed to suggest that social marketing interventions could have an impact on HIV testing rates, the authors noted the low quality of evidence and high risk of bias within the included studies [9]. A recent evidence review for the NICE Guideline on HIV testing sought evidence relating to the cost effectiveness of interventions which increase awareness, the offering and uptake of HIV testing. The review identified just two recent RCTs examining the effectiveness of mass media and communication interventions on increasing HIV testing [10]. Both studies were conducted in the US, with women, and provide only moderate evidence of effectiveness in increasing HIV testing uptake [11, 12]. Our own evaluation of a mass media intervention for GBMSM suggested partial support for the role of such interventions in improving sexual health (men with mid or high intervention exposure were more likely to have tested for HIV in the previous 6 months) [22], but recognised the limitations of such mass media interventions if run without the nuanced targeting that social marketing approaches advocate. Indeed, this is an important point and while mass media interventions might not always draw on social marketing techniques, social marketing is recognized as a theoretical approach that can effectively change behaviour [13]. A previous review found that few studies have fully incorporated social marketing criteria and there is a need for more rigorous research designs and detailed process evaluation work to identify the social marketing intervention components that are most effective [9], as well as to account for changes in technology and media use in the interim. As a result it can be challenging to identify effective components of interventions; i.e. what works, why, for whom and in what circumstances. Addressing these questions is essential in developing effective interventions.

We conducted a systematic review of the effectiveness of social marketing and mass media interventions for HIV testing in MSM published or included in systematic reviews since 2010 (updating the last published systematic review [9], from which two of the three original papers were again included [14, 15] 1 to identify the best quality evidence to guide the development of an evidence-informed, theoretically-based, social marketing intervention to increase regular HIV testing among GBMSM. The review was commissioned by the health provider NHS Greater Glasgow & Clyde (NHS GGC) to inform the development of an intervention with a clear behavioural target, clear audience segmentation and appropriate behaviour change techniques. With regard to behavioural domain, NHS GGC recognised the need for a continued focus on regular and frequent HIV testing, stressing the benefits of knowledge of HIV status. They also wanted to support men (both population wide and as individuals) to be more open in their conversations about testing, re-testing and HIV status in order to inform sexual decision-making. This project was the first step in working with NHS GGC to develop an evidence-informed social marketing intervention targeting MSM in relation to regular HIV testing.

## Methods

This review was registered on the PROSPERO International Prospective register of systematic reviews (CRD42017053451) and is reported in accordance with the Preferred Reporting Items for Systematic Reviews and Meta-Analysis (PRISMA) statement. The protocol is available at https://www.crd.york.ac.uk/PROSPEROFILES/53451_PROTOCOL_20180622.pdf.

### Search Strategy

CINAHL, Embase, Medline, PsychInfo and Web of Science were searched for articles published between 2009 and 15th November 2016 with similar and standard MeSH search terms for HIV, MSM and social marketing/mass media interventions used previously [9]. An example of the search strategy applied to Medline is presented in Supplementary File 1. No restrictions were applied to the searches in terms of language or publication type at this stage. In addition to database searches, reference lists of included articles were searched manually and relevant abstracts were checked for inclusion criteria.

### Study Selection

**Fig. 1:**
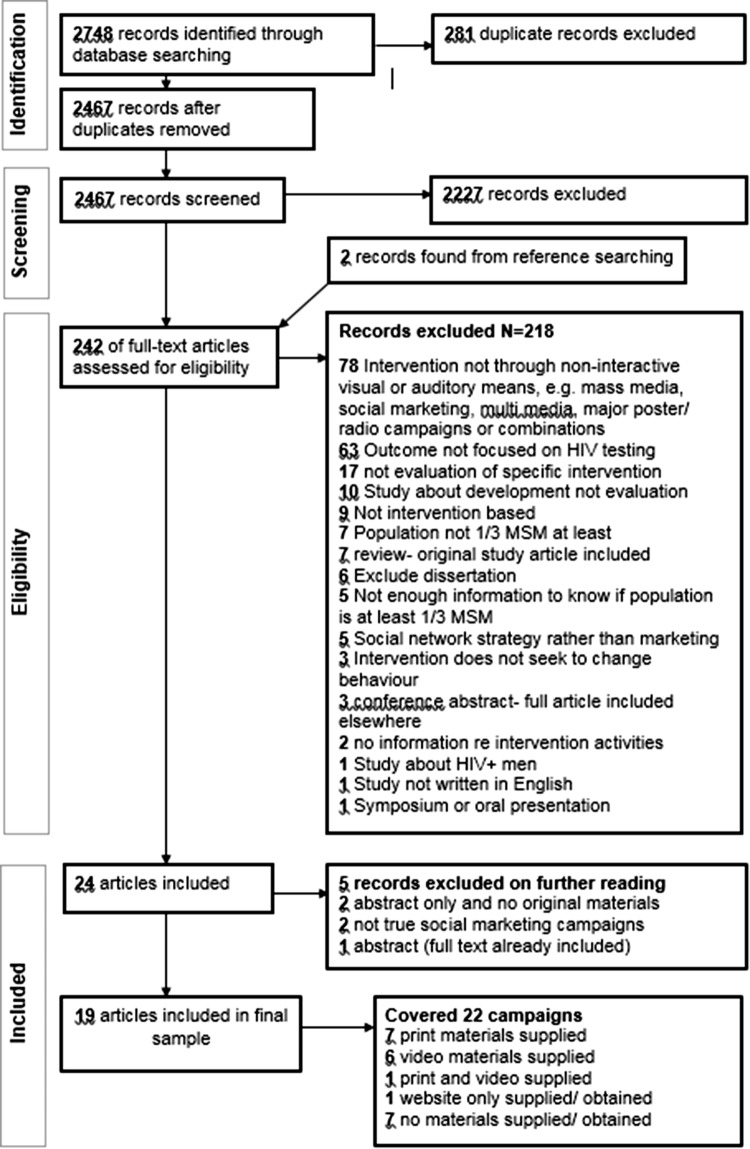
Prisma flow chart for study selection

Only studies written in English were included. Results were downloaded into, and de-duplicated in, a database in Endnote 7 (Thomson ResearchSoft). Inclusion and exclusion criteria (Table 1) were applied to screen titles and abstracts by one researcher (A2), with a 10% sub-set validated by another (A4). The inclusion criteria were informed by the rationale for the overall study and framed to the behaviour (HIV testing), target population (MSM), and the type of intervention to be developed (i.e., social marketing/mass media). Where consensus could not be reached regarding inclusion, a third reviewer was used (A1). Full reports of the selected studies were screened using the same process as before. The full inclusion/exclusion process is outlined in Fig. 1.

**Table 1:** Inclusion and exclusion criteria

	Inclusion criteria	Exclusion criteria
Design	All study types including trials, cross-sectional designs and qualitative process evaluation and qualitative studies (using in-depth interviews, focus group discussions and document analysis	No studies excluded by study design
Population	Studies in which MSM constitute at least one-third of the study sample or were specifically targeted by the intervention	Interventions where MSM constitute less than one-third of study sample
Intervention	All interventions that seek to change behaviour through non-interactive visual or auditory means. Including mass media, social marketing, multimedia, major poster and leaflet and radio interventions and combinations of the above	Intervention development without evaluation Interventions focused on social networks Interventions that do not seek to change behaviour
Comparators	Studies without comparators were included	No studies were excluded based on comparators
Context	All intervention materials must be in English, Spanish or Italian and/or have English translation attached to materials	Interventions materials only available in languages other than English, Spanish or Italian and/or had no English translation attached to materials
Outcome	Increase/decrease/no change in number and rates of HIV tests Increase/decrease in self-reported HIV tests	No reporting of HIV testing rates AND/OR self-reported testing
Publication	Published between 2010-15^th^ November 2016 (date of search) Original studies included in reviews between search dates indicated above Conference proceedings with available intervention materials (e.g. included in presentations/supplied on request)	Dissertations Conference proceedings without available intervention materials

### Data Extraction

Data extraction tools were piloted on 10% of the sample and reviewed by all authors. We extracted data on: study identifier (first author, location, year); study design (study type, method of recruitment, duration of follow-up); outcome measures (details of the specificity of the HIV testing domain were also recorded); participant details (number of participants, age); and results. Data extraction was completed by one researcher (A2) with a 10% sample validated by another (A4), and discrepancies and disagreements resolved through consensus or through discussion with other co-authors (A1/A3/A6).

### Quality Appraisal

Included studies were quality appraised using standard checklists to identify potential bias [17]. Each paper was assigned a grading for internal and external validity using standard NICE appraisal checklists for risk of bias [17], from high internal validity (all or most of the quality checklist criteria fulfilled) to low internal validity (few or no quality checklist criteria fulfilled). Studies were appraised by one researcher (A2), with a 10% sample validated by another (A4). Four of the included studies did not present sufficient detail for assessment (three were conference proceedings and one presented only illustrative examples of social marketing interventions). Papers were not excluded on the basis of quality.

### Analysis

A meta-analysis was not conducted due to the heterogeneity of the study outcomes, designs, methods and samples of the included studies. Instead, a narrative approach was used to analyse the data. The narrative synthesis is supported by summaries of the data extraction in Table 2, which outlines the key characteristics of each study included in the review. Findings of each study are presented in Table 3.

**Table 2:** Summary of included studies

Study design	Reference	Purpose (aim and objectives)	Recruitment and data collection methods	Sample	Eligibility criteria	Exclusion criteria
RCT	Tang et al. [34] China	To compare the effectiveness of a crowdsourced intervention versus a health marketing intervention to promote first time HIV testing among men who have sex with men (MSM) and transgender individuals in China	Online banner advertisement recruitment. Individuals were screened for eligibility, enrolled, and completed the survey then randomly assigned to either watch the crowdsourced video or the health marketing video. Follow-up text message 3 weeks after survey completion asking about HIV test uptake and test result	Total = 721 crowdsourced intervention = 352; health marketing intervention = 369	Born biologically male, having had anal sex with a man at least once, ≥ 16 years, never tested for HIV, provide valid mobile number	Duplicated mobile numbers were excluded
	Blas et al. [32] Lima, Peru	To study the association between video-based online interventions and proportions of HIV testing in gay-identified and non-gay identified MSM	Online banner advertisements to redirect to study website. After consent, participant randomly assigned to condition using computer algorithm. Baseline assessment, matched emails to those attending clinic	Total = 459, non-gay identified, 97 = video intervention, 90 = control (text) intervention; gay-identified, 142 = video intervention, 130 = control (text) intervention	(1) ≥ 18 years, (2) male and report having had sex with men, (3) be a resident of Lima, Peru, (4) answer the survey from Lima, Peru (5) HIV test over 12 months ago, (5) have a valid email address and, (6) do not report being HIV positive	Excluded 937 (916 did not meet criteria, 21 did not want to participate) leaving final sample of 459. Report only results from the gay and non-gay identified MSM group
	Hirshfield et al [26] United States of America	To assess the feasibility and efficacy of implementing an online intervention (videos/HIV prevention webpage) versus a no-content control	Online banner advertisements with additional email sent to US members of one of the websites. Online self-complete questionnaire at baseline and 60 days post baseline follow-up. Participants randomly assigned to conditions	Convenience sample: Total = 3,092: Control = 609 Prevention webpage = 609, Dramatic video only = 625, Documentary video only = 633, Both videos = 616	(1) identify as male; (2) ≥ 18 years; (3) live in the US.; (4) provide valid email; (5) report oral or anal sex with a current male partner (new or not), and oral, anal, or vaginal sex with at least one new partner (male or female) in the previous 60 days;( 6) ability to read/respond in English	(1) lived outside of the US; (2) identified as female, female-to-male transgender or male-to-female transgender. Duplicate cases were identified and excluded
BAstudy/Pre-Post study	Prati et al. [33] Italy	To investigate the effect of intervention on performance of HIV/AIDS protective behaviours	*General population*: computer assisted telephone survey, random digit dialling. Used Proportional quota sampling. Contacted again after 6 months. *MSM participants*—e-mail lists and Web-based communities. Self-administered anonymous online survey, again contacted again after 6 months. *Migrant participants***—**three survey sites: workplace, migrant shelter/camp, and centre for the teaching of Italian as a second language. Self-administered anonymous paper and- pencil survey and again after 6 months	General population (n = 858), MSM (n = 109), and migrants (n = 211)	≥18 years. Took part in both pre/post surveys and sexually active in the previous 6 months	Not sexually active in the previous 6 months before each interview
	Chiasson et al. [24] United States of America	To compare HIV disclosure three months before and after viewing intervention video	Online banner advertisements; online self-complete questionnaire at baseline and 3 months follow-up	Convenience sample: Original sample of 3052, reduced to 442 in final sample following drop out/inclusion criteria	Limited to the 442 men who reported sex in both baseline and follow-up interviews	Not reported
Cross sectional study	Flowers et al. [22] Glasgow, Scotland	To understand the extent of self-reported exposure to intervention among men frequenting venues for gay MSM. To explore whether sexual health related behaviours varied by degree of exposure to the intervention	Men recruited from seven bars frequented by gay men and other MSM in Glasgow ten months post intervention launch	Convenience sample: 1313 men were approached and 822 participated, Final sample = 784 post exclusions	All men present or entering the venue were approached to complete a questionnaire	Final sample excluded men who identified themselves as HIV positive
	Pedrana et al. [29] Victoria, Australia	To assess intervention impact using four key indicators: intervention awareness, HIV/STI knowledge, health seeking behaviour and HIV/STI testing	*Cross sectional data* Multiple recruitment methods: convenience samples e.g. gay community venues, gay community events; participants from a recent community- based HIV prevalence study and snowballing. Completed online surveys, linked with unique code to allow matching, surveyed at regular intervals (3-6 monthly). *Clinic data* routinely collected data from Victorian Primary Care Network for Sentinel Surveillance	*Cross sectional data* Sample of 295 gay men *Clinic data* data from 3 clinics	Men, ≥ 18 years, self-identified as gay or homosexually active in the past 5 years. Men had to have been recruited between September 2008 and April 2009 and completed any of the 3 survey rounds	Not reported
	Wilkinson et al. [30] Victoria, Australia	To explore the effectiveness of DDU to increase HIV, syphilis, gonorrhea, and chlamydia testing among MSM	*Survey data* Surveyed annually between September 2008-August 2014. Recruitment sites varied over time, included gay venues and community events, gay sporting clubs, gay online dating sites, social media, and snowballing. *Surveillance Data* The Victorian Primary Care Network for Sentinel Surveillance (VPCNSS) gathered during specific periods	1228 MSM (survey 4: n = 389, survey 5: n = 743, survey 6: n = 343, survey 7: n = 353, survey 8: n = 328). (242 included in final sample)	Males, self-identifying as Gay/MSM, ≥ 18 years, completing 3 + surveys between December 2010 and August 2014	*Evaluation cohort* recruited pre December 2010, completed less than three surveys, self-reported HIV positive. *Surveillance data* Tests within 30 days of a previous test and those indicated for HIV post-exposure prophylaxis
	McOwan et al. [15] England, UK	To evaluate the effect of an HIV testing intervention specifically aimed at gay men in central London, UK who were South European Origin, Black Origin or aged under 25 years old	Convenience sample: MSM testing for HIV within one of three London clinics during 2000, lab records were located for those matching three target groups (South European origin, Black origin, ≤ 25 years)	three clinics in London- 1999 = 65 (target clinic), 239 (other clinics); 2000 = 292 (target clinic), 236 (other clinics)	MSM testing for HIV at one of three target clinics during a specific timeframe, specifically South European origin, Black origin, ≤ 25 years	Not reported
	Guy et al. [14] Victoria, Australia	To measure the extent of any change in the uptake of testing for HIV and STIs during and subsequent to the intervention	Three types of data: *Sentinel surveillance data***—**five clinics referred to within intervention. *Routine laboratory data*—four clinics (pre, during and post intervention). *Behavioural survey*-subset of existing national survey, mainly administered at gay scene event. Surveys for 2004, 2005 and 2006 were compared	Convenience samples: those attending clinics (sentinel data/lab data), men completing Melbourne Gay Community Periodic Survey living in Victoria (numbers not explicitly stated)	*Lab/Sentinel surveillance data***—**men attending clinic within set timeframes. *Behavioural survey***—**only information from Victorian residents was included	Not reported
	Gilbert et al. [31] British Columbia, Canada	To describe the impact of targeted NAAT on identification of AHI and discuss the potential of social marketing interventions to optimise detection among MSM	Samples were included from 6 study clinics if sex recorded as male, transgendered or missing and were ≥ 18 years	Convenience sample: Testing rates from 6 clinics	sex recorded as male, transgendered or missing and were ≥ 18 years	Not reported
	Hilliam et al. [23] Scotland, UK	To evaluate the impact on awareness of HIV, attitudes towards testing, prevention and safer sex in both MSM and Health Professionals	Internet recruitment. Websites contained link to online survey. Self-reported online survey pre intervention (April–May 2010) and post intervention (October–November 2010). Post intervention recruitment added use of Grindr	Convenience sample: Pre-stage sample: 309 (MSM = 88; HP = 221) Post- stage sample: 980 (MSM = 775, HP = 205)	Not reported	Men who have sex with women only
	James [20] England, UK	To evaluate effectiveness of English intervention which promotes testing to men who have sex with men (MSM) and Africans	Limited information: Data from testing centres and community surveys	not explicitly stated	Not reported	Not reported
Retrospective cohort study or Cross sectional study	Erausquin et al. [25] Los Angeles County, USA	A pilot intervention to increase awareness of free testing services, provide incentives for getting test results, and improve access to treatment in Latino males	Community venues: outreach volunteers distributed cards target population to encourage testing. Routinely gathered data from clinic with addition of information of outreach card. Data from the intervention period (August–October 2004) compared to data from two comparison periods: May–July 2004 and August–October 2003	Convenience sample: Males testing for MSM within LAGLC’s Service, Prevention, Outreach, Treatment centre in West Hollywood-Fall 2003- n = 86, Summer 2004 n = 97, Fall 2004 n = 95	Results are limited to males who attended HIV testing within specific timeframes, ≤ age 25, reporting sexual activity with a male	Not reported
**Non-comparative study**	Brady et al. [18] England, UK	To pilot a national, free at the point of use home HIV sampling service	Testing rates were gathered during the intervention period	9,868 tests were requested over the pilot period and 6,230 (63.1%) were returned	Not reported	Not reported
	West et al. [15] England, UK	To review advertising strategies used and numbers of clients who requested POCT during NHTW	Grindr advertisements within 5 miles of clinics contained link to website including a video demonstrating POCT. Electronic records of those attending for POCT and activity data from software clinic	43 asymptomatic attendees	Not reported	Not reported
Interrupted time series	Hickson et al. [19] England, UK	Longitudinal survey to examine patterns of HIV testing and assess whether testing rates were associated with intervention periods	Internet recruitment. Invite to enrol sent to those completing a previous survey and users of two gay-dating websites. Self-reported baseline survey followed by 13 monthly follow ups	There were 3386 enrolments, following exclusions/drop outs final sample of 2047 participants	Male; England resident; ≥ 16 years; sexually attracted to/has sex with men; valid email address	Those with existing HIV-positive diagnosis and those with no or inconsistent HIV test results
	Solorio et al. [27] Seattle, USA	To assess intervention feasibility and identify processes that worked and those that did not	Convenience sample: recruited from various sites, including community events, the Internet, STD clinics, entertainment venues, and Latino newspapers and referral of peers to study. Survey every 3 months, starting with 3 months before intervention (baseline interview), 3 months into intervention and 2 months post-intervention. Self-reported questionnaires	pre-intervention assessment-50, mid-intervention assessment-44, follow-up post-intervention-41	(a) self-report Latino heritage; (b) speak Spanish; (c) biological male; (d) report sex with men in past 12 months; (e) 18-30; f) negative HIV serostatus (if known)	Not reported
**Case study/illustrative example**	Thackeray et al [28] USA	Provided illustrative example of the use of Social marketing theory in two case study interventions	Two case studies; illustrative example using social marketing theory on HIV testing intervention	two examples	Not reported	Not reported

Study design	Reference	Nature of intervention(s)	Control intervention	Outcome measures	Internal validity	External validity
RCT	Tang et al. [34] China	The 1 min video depicted 2 Chinese men embarking on a relationship and testing for HIV together. The 1 min health marketing video used a cartoon storyline to provide HIV education and promoting HIV testing	Noninferiority design without a control group	self-reported first-time HIV testing	**+**	**+**
	Blas et al. [32] Lima, Peru	Videos framed within Health Belief model and aimed to identify strategies to overcome reasons for not testing specific to target audience	Text used in control condition came from existing intervention to increase testing in Mexico	Intention to get tested, HIV testing	**+**	**–**
	Hirshfield et al [26] United States of America	Five study conditions: (1) dramatic video; (2) documentary video; (3) both videos; (4) prevention webpage; and (5) control (i.e., received no intervention content). *The Morning After***-**drama (9 min) depicting 3 gay male friends, one of whom thinks he had unprotected sex with an HIV-positive man while intoxicated and seeks advice from friends. *Talking About HIV***—**documentary (5 min) HIV positive men discuss their experiences, uses footage from a feature-length documentary, ‘‘Meth.’’	Control received no content	Self-reported HIV disclosure and other risk behaviours	**+**	**+**
BAstudy/Pre-Post study	Prati et al. [33] Italy	*United Against AIDS* (December 2012, 2 weeks; February–March 2013, 2 weeks) **-** television and radio public service announcements, print materials (e.g., posters, brochures), Web based advertisements, and cinema and newspaper advertisements. Emphasizing benefits and advantages of safer sex behaviour and getting an HIV test	Not applicable	Self reported exposure to the intervention, recent (in the previous 6 months) HIV risk behaviours and lifetime HIV testing	**+**	**++**
	Chiasson et al. [24] United States of America	The Morning after-Use of 9 min dramatic video to prompt critical thinking about HIV disclosure, HIV testing, alcohol use and risky behaviours	Not applicable	Self-reported HIV disclosure and other risk behaviours	**–**	**+**
Cross sectional study	Flowers et al. [22] Glasgow, Scotland	Social marketing intervention aimed at MSM promoting use of condoms and water-based lubricant during Anal intercourse; regular sexual health check-ups and HIV testing at least every 6 months. Materials included posters, electronic images and leaflets, with a intervention website. Posters and leaflets were distributed to both clinical and community (wider and gay scene) settings	Not applicable	Self-reported recency of HIV testing, recency of STI testing, Intention to HIV test and correct use of lubricant	**++**	**+**
	Pedrana et al. [29] Victoria, Australia	*Drama Down under*: Intervention aimed to increase access to treatment, increase awareness and knowledge; and minimize the transmission of HIV/STIs in MSM. Used print and radio advertisement, printed resources, outdoor advertisements, public events, and banner advertising on gay dating sites, ‘novel’ intervention resources (e.g., fridge magnets, drink holders, and underwear) and intervention-specific events (e.g., the “Drama Down Underwear” Show)	Not applicable	Self-reported Awareness of intervention, HIV/STI knowledge, Testing in past 6 months, Health seeking behaviours. Clinic data- testing rates	**++**	**+**
	Wilkinson et al. [30] Victoria, Australia	*Drama down under* aimed to improve screening rates and knowledge of HIV/STIs, and to reduce HIV/STIs transmission among MSM. Intervention was focused on ‘inner metropolitan Melbourne’ and included outdoor media, digital media (e.g., banners on dating Web sites), and print gay media, supported by a range of intervention material (e.g., postcards, pamphlets, fridge magnets, and underwear)	Not applicable	Self-reported HIV test in the previous 12 months, number of partners, sex with casual partners, reporting condomless sex with casual partner, recall of intervention and its message. Surveillance Data: HIV/STI monthly testing rates	**++**	**+**
	McOwan et al. [15] England, UK	*Gimmie 5* *min* (12 weeks): Advertisements in free paper distributed on the gay scene in London, images were chosen to reflect target groups	Not applicable	Testing rates at target clinic, UAI since last test, testing as result of an advert	**+**	**+**
	Guy et al. [14] Victoria, Australia	‘Check-It-Out’ targeted MSM including specific groups (community/non community attached and ‘culturally and linguistically diverse’). Intervention aimed to increase HIV and STI testing, increase regular HIV and STI testing and promote general sexual health	Not applicable	**Lab/sentinel data:** number of tests conducted per month. *Behaviour study* changes in self-reported testing patterns	**+**	**–**
	Gilbert et al. [31] British Columbia, Canada	1) What Are You Waiting For - focused on raising awareness of rapid testing and NAAT (December 2009 to February 2010) 2) Hottest At The Start- focused on raising awareness of AHI and increased transmission risk in MSM in new relationships or engaging in risky sex.(June to August 2011)	Not applicable	Testing rates of those attending clinic	**–**	**–**
	Hilliam et al. [23] Scotland, UK	HIV Wake up Intervention (May 2010)- to inform MSM across Scotland about HIV and levels of transmission, the benefits of prevention and regular testing and where they can go to seek more information and advice. Resources included leaflets and posters, digital online banners and targeted web pages and other web media (e.g. emails targeted at Gaydar users). Materials displayed in ‘scene’ venues and wider community	Not applicable	Self reported knowledge and understanding around HIV testing, awareness and exposure to intervention, HIV testing and other risk behaviours	**–**	**+**
	James [20] England, UK	National HIV Testing week (four weeks) promoted through targeted print, social media and outdoor advertising. Stakeholders also provide expanded testing services	Not applicable	Clinic based testing rates	*Not assessed- insufficient detail*	
Retrospective cohort study or Cross sectional study	Erausquin et al. [25] Los Angeles County, USA	Outreach cards provided at Latino-oriented gay club and event nights could be swapped for a movie pass at the time of testing. Information was also advertised on two Internet sites and in three gay/bisexual-oriented magazines. Again these included outreach cards that could be exchanged for movie passes at the time of testing	Not applicable	Testing rates of those attending clinic	**–**	**+**
**Non-comparative study**	Brady et al. [18] England, UK	HIV testing interventions and social media marketing were used to increase HIV testing rates, in particular those requesting self-tests	Not applicable	Testing rates	*Not assessed- insufficient detail*	
	West et al. [15] England, UK	Grindr users within 5 miles, received link to website with POCT video, Poster interventions were also in place at the time	Not applicable	Clinic based testing rates and number of visits to website	*Not assessed- insufficient detail*	
Interrupted time series	Hickson et al. [19] England, UK	1) ‘I Did It’ (December 2010-April 2011)-Terrence Higgins Trust (THT) intervention aimed to make MSM aware of ease and convenience of HIV testing. Used media advertisements, radio and website. 2) ‘Clever Dick/Smart Arse’ (November 2011-February 2012)-THT intervention promoting condom use (3)‘Count Me In’- GMFA, encouraged men to commit to an action plan which included HIV testing	Not applicable	Self reported HIV testing behaviour and self reported exposure to interventions	**++**	**+**
	Solorio et al. [27] Seattle, USA	*Tu Amigo Pepe* Spanish-language radio PSAs, a Web site, social media outreach, a mobile phone reminder system, print materials, posters in stores frequented by Latinos, and a free hotline	Not applicable	Self reported HIV testing rates, intention, experiential attitude, instrumental attitude, self-efficacy, and norms toward HIV testing	**+**	**–**
**Case study/illustrative example**	Thackeray et al [28] USA	One on mental health, second *You Know Different***—**large-scale intervention focused on increasing HIV testing among African American youth	Not applicable	HIV testing rates	*Not assessed- insufficient detail*	

**++ **All or most of the checklist criteria have been fulfilled, where they have not been fulfilled the conclusions are very unlikely to alter (high internal validity)

+ Some of the checklist criteria have been fulfilled, where they have not been fulfilled, or not adequately described, the conclusions are unlikely to alter (medium internal validity)

− Few or no checklist criteria have been fulfilled and the conclusions are likely or very likely to alter (low internal validity)

**Table 3:** Summary of included study results and intervention effectiveness

Study	Primary results (for MSM only)	Intervention had a negative effect (i.e, decrease in uptake of HIV testing)	Intervention had no effect	Intervention had an effect on the antecedent of behaviour (e.g. intentions to test or knowledge)	Indicative of some positive desired behaviour change	Indicative of clear behaviour change in desired direction
Blas et al. [32] Lima, Peru	In the non-gay identified group, participants in the video group were more likely to report intentions of getting tested in the next 30 days (RR = 2.77, 95% CI 1.42–5.39), make an Internet appointment (RR = 1.48, 95% CI 1.13–1.05) and to attend the clinic for testing (11.3% versus 0%, p-0.001) than participants in the text-based intervention In the gay identified group, differences in the reporting of intentions of getting tested for HIV within the next 30 days (RR = 1.54; 95% CI: 0.74–3.20), in making an Internet appointment (RR = 1.11; 95% CI: 0.88–1.39) and in attending the clinic for HIV testing (RR = 1.07; 95% CI: 0.40–2.85) were not statistically significant between participants from the video-based intervention and the text-based intervention				**X**	
Brady et al. [18] England, UK	8015 self-sampling kits were requested by MSM during the pilot period, with 65.2% returned, with a positivity rate of 1.6%. Authors report the increase in requests for tests was “strongly linked to HIV testing interventions and marketing the service on social media”, but no results are provided, other than that a single Grindr message resulted in 3575 visits to the online order page				**X**	
Chiasson et al. [24] United States of America	HIV testing was reported by 120 men, but differences in how data were collected at baseline and follow up did not allow for comparison of testing between the two time points		**0**			
Erausquin et al. [25] Los Angeles County, USA	MSM clients testing in the intervention period were younger (F(2,233) = 3.13, p = 0.045) and more likely to report being Latino than clients in the non-intervention period (× 2(2) = 8.33, p = 0.021)				**X**	
Flowers et al [22] Glasgow	When adjusted for age, area of residence and use of the gay scene, those with high intervention exposure were more likely to have tested for HIV in the previous 6 months than those with no exposure (AOR = 1.96, 95% CI 1.26-3.06, p = 0.003), although causality cannot be addressed					**X**
Gilbert et al [31] British Columbia, Canada	The volume of HIV tests at study clinics increased over the post-implementation period (p = 0.023) and there was an increase in acute and non-acute HIV diagnosis rates and an increase in the acute to non-acute rate ration (p = 0.015) at study sites with the second social marketing intervention. *CIs not provided*					**X**
Guy et al [14] Victoria, Australia	The sentinel surveillance network showed no increase in the overall extent of HIV testing and no difference in the proportion of MSM reporting regular annual HIV testing during the intervention (43%) and post intervention (41%). Between 2004 and 2006, the annual behavioural surveys showed only a slight increase in the overall proportion on MSM reporting having an HIV test in the last 12 months (2004 = 60.3%, 2005 = 61.4%, 2006 = 61.9%; χ^2^ = 0.34) *CIs not provided*		**0**			
Hickson et al [19] England, UK	The association between awareness of the intervention and HIV testing weakened after adjusting for age-group, SHA of residence and relationship status, sexual partners and testing history, and exposures to other health promotion interventions (rate ratio 1.11, 95% CI 0.85 to 1.45, p = 0.45)				**X**	
Hilliam et al [23] Scotland, UK	Those aware of the intervention were more likely to have been tested in the last 6 months: Gaydar Sample: Aware = 33%; Not Aware = 16%; Non-Gaydar Sample: Aware = 38%; Not Aware = 9% (*CIs not provided*). Although testing in last 6 months is higher for those aware of the intervention, intervention awareness may not be the cause of the testing activity, as the act of obtaining a test may have led to intervention awareness (e.g. intervention posters may have be seen at the testing site)					**X**
Hirshfield et al. [26] United States of America	Among HIV-negative and untested men who completed follow-up (n = 1,116), 21% reported getting an HIV test; however there were no differences across study conditions or changes in HIV testing observed in any of the conditions. (Pooled videos OR = 1.33, CI 0.99-1.81; Webpage Behavior Change OR = 1.40, CI 0.76-2.62; No-Content Control Behavior Change OR = 1.35, CI 0.73-2.54)		**0**			
James [20], England, UK	Promotion of NHTW led to 8,464 home sampling HIV tests being ordered in the two weeks leading up to and during NHTW compared to 618 orders in the three weeks before, but no data are presented on tests returned				**X**	
McOwan et al. [15], England, UK	Number of MSM HIV testing at the intervention clinic rose from 65 in 1999 to 292 during the intervention, with a proportionately greater rise in the three groups targeted by the intervention, but no change in the total number of MSM tested at two comparison clinics for HIV during the intervention. The proportion stating that HIV testing uptake was in response to an advertisement, poster or leaflet increased from 1/65 in 1999 to 162/292 after the intervention (p = 0.001). *CIs not provided*					**X**
Pedrana et al. [29] Victoria, Australia	HIV testing rates increased during the initial intervention period (17%, p = 0.01), and during the continued intervention period (27%, p = 0.01), compared with the pre-intervention period. *CIs not provided*					**X**
Prati et al. [33] Italy	For MSM participants, the probability of undertaking HIV test did not change in the exposed (χ2(1) = 3.20, p = .074, r = .23; R = 4.63, F = 5.00, p = .063) and the unexposed subsample (χ2(1) = 0.00, p = .999, r = .00; R = 1.00, F = 1.00, p = .999)		**0**			
Solorio et al. [27], Seattle, USA	From pre-intervention to mid-intervention, there were increases in intention to test (b = 1.1, 95% CI 0.3-2.0, p = 0.01), attitudes to testing (b = 0.4, 95% CI 0.2-0.5, p = 0.001) and average self-efficacy towards testing (b = 0.3, 95% CI 0.1-0.4, p = 0.004) and average injunctive norms to testing (b = 0.3, 95% CI 0.1.6, p = 0.01) No increase in HIV testing rates (OR 1.7, 95% CI 0.9–3.4, P = 0.1)			**X**		
Tang et al. [34] China	In the crowdsourced intervention arm, 114 of 307 (37%) reported testing for HIV compared with 111 of 317 (35%) in the health marketing arm. For the complete case analysis, the estimated difference in proportions between arms was 2.1% (95% CI, −5.4% to 9.7%). Using multiple imputation, the estimated difference in proportions was 3.1% (95% CI, −4.5% to 10.7%). *Significance values were not provided.* Participants who watched the crowdsourced video more than once were more likely to test for HIV compared with those who watched the crowdsourced video only once, with a risk difference of 25.8% (95% CI, 15.0%–36.7%)					**X**
Thackeray et al. [28] USA	In the pilot phase, testing increased more than 300%, and service delivery partners increased their capacity to provide culturally appropriate testing services. Testing at participating organizations increased 153% among the target populations. Nearly 90% of youth surveyed said that the intervention had an impact on their decision to seek an HIV test *(no data or statistical analyses provided)*					**X**
West et al. [21] England, UK	The average MSM number of daily visits to the website increased from 250 to 600 per day and the POCT video was viewed 126 times during testing week. 43 asymptomatic attendees requested POCT, of which 21 were MSM and 15 reported that they attended as a result of the Grindr advertisement. *(no data or statistical analyses provided)*			**X**		
Wilkinson et al. [30] Victoria, Australia	Although intervention awareness was high among 242 MSM completing 726 prospective surveys, intervention recall was not associated with self-reported HIV testing. Across surveys, between 42.6% and 53.2% of respondents correctly recalled DDU intervention messages, with authors reporting a moderate decline in DDU message recall between first (T-2) and most recent surveys (T0) (P = 0.49). Contemporaneous and lagged message recall was not associated with HIV testing in the 12 months before T0. The increases in the monthly testing trends for HIV and syphilis tests continued after DDU implementation, though modest. (differences in the slopes: 1.7 (−1.6 to 5.1) HIV testing observed pre- to post-DDU period suggests a continuation of trends rather than a shift toward more frequent testing among men		**0**			

### Effectiveness

Given the heterogeneity of contributing studies, findings were assessed and categorized in terms of effectiveness as follows:

1. Intervention had a negative effect (i.e. decrease in uptake of HIV testing).
2. Intervention had no effect
3. Intervention had a positive effect on the antecedent of behaviour (e.g. intentions to test or knowledge)
4. Indicative of some positive desired behaviour change (i.e. some increase in uptake of HIV testing or in one segment of the population, but not all)
5. Indicative of clear behaviour change in desired direction (i.e. increase in uptake of HIV testing)

## Results

A total of 2748 articles were identified, with an additional two found during manual reference searches. 242 studies met criteria for full text screening from which 223 were subsequently excluded as not meeting eligibility criteria. 19 studies were included in the review (Fig. 1, Table 2).

### Study Characteristics

Of the 19 included studies, seven were conducted within the UK, with the majority of these conducted in England (n = 5) [15, 18–21] and the remainder in Scotland (n = 2) [22, 23]. Five studies were conducted in United States of America [24–28], three in Australia [14, 29, 30] and the rest conducted in Canada [31], South America [32], Italy [33] and China [34]. Twelve studies recruited participants with a total sample size of 10,894 (range 50–3092), one study did not report on sample size [14]. The average age of participants ranged from 22–47 years (nine studies did not provide an average age of participants [15, 18, 20, 21, 23, 24, 28, 31, 34]).

Nine studies were cross-sectional surveys [14, 15, 20, 22, 23, 25, 29–31], three were randomised control trials [26, 32, 34], two were non-comparative studies [18, 21], two were pre/post studies [24, 33], two were interrupted time series [19, 27] and there was one case study [28]. As a result, the majority did not contain a control group. The 19 studies evaluated 22 separate interventions. A variety of recruitment methods were adopted across the studies, with some combining more than one method (see Table 2). The majority of studies used online methods to recruit participants (e.g. banner adverts, use of online communities, direct emails to website users), eight used clinic visit data, six recruited participants in community venues (e.g. bars) and five recruited through other means (e.g. apps or peer referral). The average response rate within the studies was 48.5% (range 1.9–87.1%, median 62.6%).

The majority of studies included testing as a primary outcome (routinely-collected or self-reported testing data, with some reporting both). Just two studies explicitly measured frequency of HIV testing [19, 31], four included a measure of recency of previous HIV test [14, 22, 23, 30] whilst the remaining studies used isolated self-reported HIV testing or intention to test or testing rates at clinics within a specific time period. Additionally, five studies reported on antecedents of testing (e.g., knowledge or intentions) [22, 23, 27, 29, 32] and six reported on other outcomes (e.g. risk behaviours such as Condomless Anal Intercourse (CAI) and other HIV risk behaviours) [15, 23, 24, 26, 30, 33] (Table 3). Ten studies [14, 15, 18, 20, 21, 25, 27, 29–31] used routinely collected data (clinic samples), with a total of 73,704 tests (one study did not report actual numbers [27]). Twelve studies recruited participants [14, 19, 22–24, 26, 27, 29, 30, 32–34] with a total sample size of 10,894 (again one study did not report sample size [14]). Four studies merged participant self-reports and routinely collected data [14, 27, 29, 30], with two evaluating the same intervention at different time points [29, 30]. Six of the included studies gathered data only during the intervention period [15, 18, 20, 25, 30] whilst information was unclear about post intervention follow-up periods for four interventions [28, 29, 32, 33]. Of the nine studies reporting clear follow-up periods there was a wide variety of time frames. Only three of these studies reported post intervention follow-up periods over 6 months [14, 22, 31] whilst the remaining studies ranged from 3 weeks [34] up to 6 months [23].

### Intervention Content

The purpose and nature of the interventions are reported in Table 2. Of the 19 studies, ten included specific reference to social marketing’s theoretical principles [14, 19, 22, 23, 26–31]. Very little could be gleaned from the studies about their behaviour change focus beyond a desire to increase HIV testing. This does not mean that actual mechanisms or techniques to change behaviour were not employed (the contrary to which was evident and reported in the BCT and theory coding analyses reported elsewhere, but instead that this was often implicit in the materials employed rather than explicit in the descriptions of these). However, studies generally included detailed descriptions of the nature of the intervention, the provider and the content. Most were delivered online or in gay venues and other community settings, with none delivered via one medium alone, and most relied on a variety of delivery media, including posters, leaflets and adverts. Three studies reported that delivery was supported by outreach workers or peer educators [22, 25, 28]. Most reported use of an intervention name, brand or logo and there was a considerable mix of tone (e.g., informative, positive, humorous etc.). Interventions were delivered for up to 14 months, but the studies were less clear on intensity (i.e., the length of time potential users might engage with intervention materials). Overall, the interventions used an array of different imagery, but the majority used photographs as the central image (our visual analysis interrogated audience reading of this and the implications of it for future intervention design is reported elsewhere). All but one of the interventions [21] featured actors who could be interpreted as representative of the target audience, implicitly or explicitly identifying actors as MSM. The interventions were primarily informal and direct in tone and all also featured text of some kind, most frequently phrased as an instruction or statement to convey key messages.

### Quality Appraisal by Study Design

Whilst both internal and external validity gradings are reported (Table 2), the current study will focus on the internal quality (the robustness of the findings) rather than how generalizable the findings are (external validity). Quality appraisal was assessed for 15 studies, with no studies fulfilling all or most of the checklist criteria for both internal and external validity (see Supplementary file 2). Within the current study, four of the included studies were graded as showing high internal validity, fulfilling all or most of the checklist criteria of internal validity. Three of these studies used a cross sectional design [22, 29, 30] whilst the remaining study used an interrupted time series design [19]. Only four studies were graded as low internal validity, fulfilling none or few of the checklist criteria for internal validity, three of which used a cross sectional or retrospective cohort study [23, 25, 31] and one was a pre-post design [24]. Those that scored poorly on internal validity were largely judged to do so based on a general lack of information about the population (i.e. potential bias in sampling), lack of information around selection of participants and confounding variables (and comparison group), lack of detail regarding reliability of measures used (i.e. self-reported testing/unvalidated measures) and a lack of detail regarding other factors that may influence effectiveness of intervention (i.e. information relating to intensity of exposure to intervention).

### Effectiveness of Interventions by Study Design

An overview of results and the relative effectiveness of interventions across four categories of effectiveness is provided in Table 3. Seven studies reported results that were indicative of behaviour change in the desired direction (i.e. an increase in testing) [15, 22, 23, 28, 29, 31, 34]. An additional five [18–20, 25, 32] reported results indicative of some positive desired behaviour change (i.e. an increase in one segment of the population targeted but not all [32]); an increase in the proportional representation of target populations in clinic samples [25]; or an increase in requests for self-sampling HIV tests [18, 20]. In one study, the increase in HIV testing was no longer statistically significant after adjusting for key demographics, sexual and testing history, and exposure to other health improvement interventions (rate ratio 1.11, 95% CI 0.85–1.45, p = 0.45) [19]. Two studies reported that the intervention had an effect on the antecedents of behaviour (e.g. knowledge of, or intentions for, testing) [21, 27] and the final five reported that the intervention had no effect [14, 24, 26, 30, 33]. None of the included studies reported a negative effect.

Looking at effectiveness in relation to study design, of the three RCTs included within the current study, one was indicative of clear behaviour change in the desired direction [34], one was indicative of some positive desired behaviour change [32] and the final study showed no effect [24]. All were graded as medium internal validity, fulfilling at least some but not all of the checklist criteria for internal validity.

Of the eight cross sectional or retrospective cohort studies, one study which was indicative of clear behaviour change in the desired direction was graded as high internal validity [22]. The final two studies graded as high internal validity used different analytical techniques and timescales to assess the impact of the same intervention (Drama Down Under) with different results [29, 30]. The first suggested that there was some initial evidence of an increase in testing across the duration of the intervention) [29], but the latter, when incorporating insights from more recent data sets, concluded that the increase in HIV testing suggested a continuation of temporal trends rather than more frequent testing among men [30].

Two of the cross sectional studies were graded as medium internal validity [14, 15], although the results for these were mixed with McOwan et al. suggesting results indicative of clear behaviour change and Guy et al. suggesting no effect. Three cross sectional or retrospective cohort studies were graded as low internal validity and yet reported results indicative of clear behaviour change [23, 31] or some positive desired behaviour change [25]. The final cross sectional study was unable to be assessed for internal validity due to insufficient study detail, although their results did indicate some positive desired behaviour change [20].

The two pre-post studies included showed no intervention effect, although internal validity varied with the first graded as medium internal validity [33] and the second graded as low internal validity [24]. Both studies using the interrupted time series design had results indicative of some positive desired behaviour change [19] or an effect on the antecedents of behaviour [27]. These studies were both graded positively in terms of internal validity with the Hickson et al. graded as high internal validity and Solario et al. graded as medium internal validity.

Studies using the non-comparative design were unable to be assessed for internal quality due to insufficient reported methodological detail, although their results did indicate some positive desired behaviour change [18] or an effect on the antecedents of behaviour [21]. Finally, the current study was unable to assess the internal validity of the Thackeray et al. case study (2011), although their results were indicative of clear behaviour change in the desired direction.

## Discussion

**Fig. 2:**
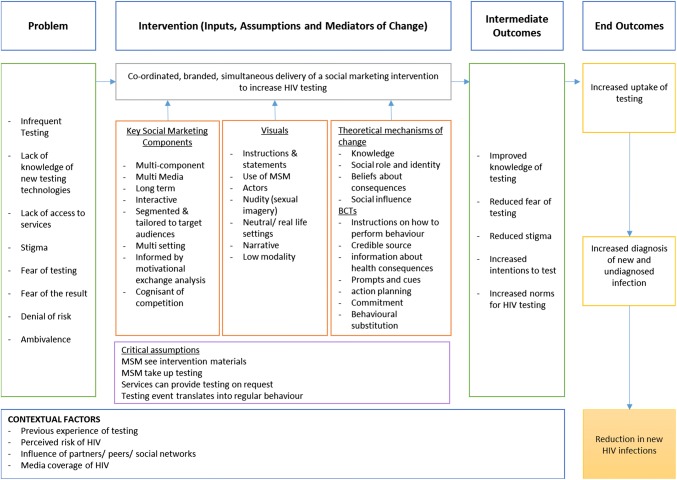
Logic model for an evidence-informed, theoretically-based, social marketing intervention to increase regular HIV testing among GBMS

This systematic review has examined the effectiveness of contemporary social marketing and mass media interventions for HIV testing with GBMSM. Our review has demonstrated that there is now a growing body of evidence for the effectiveness of social marketing and mass media interventions to increase HIV testing among GBMSM. However, there was heterogeneity of interventions, study quality was mixed and few have adopted the most rigorous study designs. Of seven studies reporting an increase in HIV testing, five were cross sectional studies (two graded as high internal validity, one medium and two low internal validity), one was an RCT (medium internal validity) and one case study (unable to be assessed for validity). This speaks to the challenge of evaluating this particular type of intervention. Within the context of the limitations of general effectiveness reviews, we need to know what works, for whom, when and how. Further details relating to the specific content of the interventions can be found in forthcoming papers relating to an analysis of mechanisms of change [35] and social marketing and visual design components of the interventions [36]. By reviewing the key processes involved in mass media consumption, and examining the role of theory and behaviour change techniques employed in message delivery, we have achieved a high quality integration of multi-source data from different theoretical perspectives. In this way we have optimized the potential content of social marketing interventions to increase HIV testing in evidence-based and theoretically-informed ways. A detailed logic model that sets out the key components of social marketing, visual design and theoretical mechanisms of behaviour change that the overall review has suggested are required as inputs for an intervention is shown in Fig. 2.

Our review is the first to explore patterns between study type (RCT, cross-sectional or pre/post or cohort study design), internal validity and intervention effectiveness. Seven of the 19 studies reported results indicative of an increase in HIV testing and another five reported results indicative of some positive desired behaviour change. Previous reviews demonstrated a lack of evidence on the effectiveness of social marketing and mass media interventions to increase HIV testing among GBMSM. The 2011 Cochrane review included just two studies in their final analysis [9], while the more recent evidence review for the NICE Guideline on HIV testing identified a further two recent RCTs, neither of which were conducted with GBMSM [10].

The 2011 Cochrane review called for more rigorous research designs and detailed process evaluation work to identify the social marketing intervention components that are most effective [7]. However, the studies included in this review were of relatively poor quality, with most study designs being cross-sectional and only three RCTs included. While two RCTs had results that were either indicative of behaviour change [34] or some positive desired behaviour change [32], the latter was judged to be of poor study quality. Our findings, unsurprisingly, suggest that the study designs, analytical techniques and timescales used to assess the impact of interventions can influence interpretations of effectiveness. Changes in testing rates across a population or in cross-sectional studies might not be the result of the intervention, but instead indicative of temporal trends and may be affected by a variety of other factors. This is particularly evident in the competing conclusions on the effectiveness of one intervention by two studies using different analytical techniques [29, 30]. Our review speaks to the challenge of evaluating this particular type of intervention, which has been discussed previously [22]. The lack of RCTs identified may be indicative of the difficulty of using this research design in the evaluation of population-level social marketing interventions. There is a need to consider and explore the potential for the development and use of alternatives, such as natural experiment designs, which are appropriate when evaluating population level and policy interventions [37], in order to overcome barriers associated with wide-population reach and exposure.

We found no qualitative studies or process evaluations, despite the importance of these to inform the design and implementation of future interventions. This demonstrates the need to further evaluate the social marketing and theoretical behaviour change content of interventions simultaneously. We had limited access to information regarding other factors that may have influenced effectiveness, e.g. context of delivering the intervention; knowledge of existing HIV interventions; changes to services and political/cultural setting in the locations in which interventions were delivered. Whilst we acknowledge that some of these factors may be impossible to control for within a real world setting, it is crucial that we consider this within intervention evaluations [38]. Detailed intervention development studies and accompanying process evaluations are needed in the future to enable consideration of this context in understanding intervention delivery.

### Strengths and Limitations

We have conducted a rigorous search and systematic review accompanied by a narrative synthesis, updating and adding to previous work, providing a valuable contribution to the field. Whilst one of the included interventions [22] was conducted by authors involved within this review, we are confident that the data extraction process has limited the potential bias of reviewing its effectiveness as those authors were not involved in the data extraction of that study. The overall quality of evidence was relatively low and thus our findings should be interpreted with caution. As noted earlier, this is a consequence of the difficulty in evaluating population-level social marketing interventions. Despite this, we are confident that the methods adopted in the current study have contributed to a robust synthesis of existing evidence. Neither did we conduct an analysis of the cost-effectiveness of reviewed interventions, which is an important component for future evaluations. Our review focused on mass media, social marketing, multimedia, major poster and leaflet and radio interventions and combinations of these. Review of alternative media, particularly using social media or social networking sites was beyond of the scope of the work we were commissioned to undertake, but this is worthy of further research [39, 40]. It is also important to note that all of the included studies were conducted prior to the introduction or availability of PrEP for HIV prevention. This has dramatically altered the context of HIV testing, presents issues for the transferability of previous interventions to future contexts, and will need to be considered within any future intervention development. Whilst few studies reported measures relating to frequency and/or recency of HIV testing this may reflect a more recent emphasis on increasing and measuring HIV testing frequency. Future evaluations need to factor in appropriate timeframes to allow accurate measurement of changes in HIV testing frequency post intervention.

## Conclusions

HIV testing is not a simple behavioural domain and there are important differences in the ways people think about testing and the antecedents to testing. Testing decisions for example are very different across testing scenarios such as testing for the first time, in relation to a high risk event [ [14]), in relation to regular check-ups or to access PrEP [41]. Public health gain is equally distinct. Interventions should be designed to accommodate the diverse antecedents of decisions to test. Significant knowledge gaps remain in relation to such segmentation, the means of increasing the frequency of HIV testing and on the maintenance of appropriate testing patterns over time.

To consolidate the individual and public health benefits presented by HIV testing interventions, HIV testing interventions should be considered in relation to the full range of technological, psychosocial and sociocultural contexts of HIV testing [41]. The increasing diversification and technological variation of tests available (point of care, self-sampling or self-testing) demands systematic consideration of the right test for particular circumstances and sub-populations (i.e., permutations of audience segmentation regarding for example, previous testing history, perceived likelihood of positive results). Intervention development and potential intervention content should potentially present the range of HIV tests according to individual circumstances. Despite the growing body of evidence for the effectiveness of social marketing/mass media interventions to increase HIV testing that we have demonstrated here, there remains a need for well-designed, high quality, robust and innovative evaluations, with accompanying process evaluations, to allow for better clarity in identifying the best social marketing and mass media interventions that can increase appropriate HIV testing among GBMSM.

## Electronic supplementary material

Below is the link to the electronic supplementary material.

Supplementary material 1 (DOCX 26 kb)

## Footnotes

1 The third, Darrow et al. [16], was not included because it evaluated an intervention relating to syphilis rather than HIV.
