# Supplementary material for: The Effectiveness of Social Marketing Interventions to Improve HIV Testing Among Gay, Bisexual and Other Men Who Have Sex with Men: A Systematic Review
Source: AIDS Behav. 2019 Apr 20;23(9):2273–303. doi: 10.1007/s10461-019-02507-7 (PMC6766472; doi:10.1007/s10461-019-02507-7)
Supplement: Supplementary file 1 — Supplementary material 1 (DOCX 26 kb) [file 10461_2019_2507_MOESM1_ESM.docx]

#### Supplementary File 1: Search strategy example

**Epub Ahead of Print, In-Process & Other Non-Indexed Citations, Ovid MEDLINE(R) Daily and Ovid MEDLINE(R) 1946 to Present 2.11.16**

| 1 | exp HIV Infections/ | 253789 |
| --- | --- | --- |
| 2 | exp HIV-1/ | 72027 |
| 3 | exp Sexually Transmitted Diseases/ | 308146 |
| 4 | Acquired Immunodeficiency Syndrome/pc [Prevention & Control] | 13358 |
| 5 | "human immunodeficiency virus".tw. | 77759 |
| 6 | HIV*.tw. | 271457 |
| 7 | "human immuno-deficiency virus".tw. | 215 |
| 8 | "acquired immune deficiency syndrome".tw. | 5393 |
| 9 | "acquired immuno-deficiency syndrome".tw. | 116 |
| 10 | "acquired immune-deficiency syndrome".tw. | 5393 |
| 11 | (human immun* and deficiency virus).mp | 552 |
| 12 | 1 or 2 or 3 or 4 or 5 or 6 or 7 or 8 or 9 or 10 or 11 | 420489 |
| 13 | exp Advertising as Topic/ | 13769 |
| 14 | exp Cell Phones/ | 8099 |
| 15 | exp Internet/ | 62577 |
| 16 | exp "Marketing of Health Services"/ | 16714 |
| 17 | exp Mass Media/ | 42237 |
| 18 | exp Mobile Applications/ | 1435 |
| 19 | exp Computers, Handheld/ | 3414 |
| 20 | exp Smartphone/ | 609 |
| 21 | exp Social Marketing/ | 2316 |
| 22 | exp Social Media/ | 3158 |
| 23 | exp Social Networking/ | 1779 |
| 24 | "broadcast media".tw. | 88 |
| 25 | "cell phone".tw. | 1331 |
| 26 | "chat room".tw. | 119 |
| 27 | "digital literacy".tw. | 50 |
| 28 | "gay sexual networking".tw. | 3 |
| 29 | "mass communication".tw. | 227 |
| 30 | "mass media".tw. | 4084 |
| 31 | "new media".tw. | 555 |
| 32 | "mobile device".tw. | 544 |
| 33 | "mobile phone".tw. | 3634 |
| 34 | "personal digital assistant*".tw. | 958 |
| 35 | "sexual media".tw. | 29 |
| 36 | "smart phone".tw. | 335 |
| 37 | "social media".tw. | 3802 |
| 38 | "social networking applications".tw. | 14 |
| 39 | "text messag*".tw. | 2166 |
| 40 | App.tw. | 16601 |
| 41 | apps.tw. | 2233 |
| 42 | Facebook.tw. | 1735 |
| 43 | GSN.tw. | 251 |
| 44 | Internet.tw. | 37642 |
| 45 | Iphone.tw. | 494 |
| 46 | Online.tw. | 65740 |
| 47 | PDA.tw. | 7526 |
| 48 | Smartphone.tw. | 2798 |
| 49 | texting.tw. | 477 |
| 50 | Web.tw. | 71401 |
| 51 | Website.tw. | 11939 |
| 52 | Websites.tw. | 6907 |
| 53 | media.tw. | 212557 |
| 54 | 13 or 14 or 15 or 16 or 17 or 18 or 19 or 20 or 21 or 22 or 23 or 24 or 25 or 26 or 27 or 28 or 29 or 30 or 31 or 32 or 33 or 34 or 35 or 36 or 37 or 38 or 39 or 40 or 41 or 42 or 43 or 44 or 45 or 46 or 47 or 48 or 49 or 50 or 51 or 52 or 53 | 490296 |
| 55 | exp Homosexuality, Male/ | 12035 |
| 56 | exp Bisexuality/ | 3432 |
| 57 | exp Transsexualism/ | 3182 |
| 58 | "cisgender male".tw. | 8 |
| 59 | "men who have sex with men".tw. | 7634 |
| 60 | "same sex".tw. | 5334 |
| 61 | "same-sex".tw. | 5334 |
| 62 | Bisexual.tw. | 6129 |
| 63 | Gay.tw. | 8148 |
| 64 | Homosexual*.tw. | 12701 |
| 65 | MSM.tw. | 6762 |
| 66 | Queer.tw. | 648 |
| 67 | transexual.tw. | 16 |
| 68 | transgender.tw. | 2235 |
| 69 | transsexual.tw. | 1029 |
| 70 | YMSM.tw. | 215 |
| 71 | 55 or 56 or 57 or 58 or 59 or 60 or 61 or 62 or 63 or 64 or 65 or 66 or 67 or 68 or 69 or 70 | 39854 |
| 72 | 12 and 54 and 71 | 1569 |
| 73 | limit 72 to yr="2009 -Current" | 1143 |

#### Supplementary File 2: Item-level Quality Appraisal Results

|  | **Section 1: Population** | | | **Section 2: Method of selection of exposure (or comparison) group** | | | | | **Section 3: Outcomes** | | | | | **Section 4: Analyses** | | | | **Section 5: Summary** | |
| --- | --- | --- | --- | --- | --- | --- | --- | --- | --- | --- | --- | --- | --- | --- | --- | --- | --- | --- | --- |
|  | **1.1 Is the source population or source area well described?** | **1.2 Is the eligible population or area representative of the source population or area?** | **1.3 Do the selected participants or areas represent the eligible population or area?** | **2.1 Selection of exposure (and comparison) group.  How was selection bias minimised?** | **2.2 Was the selection of explanatory variables based on a sound theoretical basis?** | **2.3 Was the contamination acceptably low?** | **2.4 How well were likely confounding factors identified and controlled?** | **2.5 Is the setting applicable to the UK?** | **3.1 Were the outcome measures and procedures reliable?** | **3.2 Were the outcome measurements complete?** | **3.3 Were all the important outcomes assessed?** | **3.4 Was there a similar follow-up time in exposure and comparison groups?** | **3.5 Was follow-up time meaningful?** | **4.1 Was the study sufficiently powered to detect an intervention effect (if one exists)?** | **4.2 Were multiple explanatory variables considered in the analyses?** | **4.3 Were the analytical methods appropriate?** | **4.6 Was the precision of association given or calculable?  Is association meaningful?** | **5.1 Are the study results internally valid (i.e. unbiased)?** | **5.2 Are the findings generalisable to the source population (i.e. externally valid)?** |
| Blas et al. 2010 | **+** | **+** | **+** | ++ | **+** | ++ | ++ | **-** | ++ | **-** | **−** | ++ | ++ | ++ | N/R | **−** | **+** | **+** | **−** |
| Brady et al 2014 | Not assessed- abstract only | | | | | | | | | | | | | | | | | | |
| Chiasson et al 2009 | N/R | **−** | **−** | N/A | - | N/A | **+** | + | **−** | **−** | - | N/A | + | N/R | + | + | + | **−** | **+** |
| Erausquin et al 2009 | **+** | ++ | - | - | + | - | - | + | + | **-** | **−** | ++ | N/A | N/R | ++ | ++ | ++ | - | + |
| Flowers et al 2009 | **+** | **+** | ++ | N/A | ++ | N/A | ++ | ++ | + | ++ | **−** | N/A | N/A | N/R | ++ | ++ | + | ++ | + |
| Gilbert et al 2013a | N/R | - | ++ | - | + | N/A | - | + | - | + | - | N/A | N/A | N/R | - | - | - | - | - |
| Guy et al 2009 | N/R | ++ | ++ | - | - | N/A | - | + | - | + | - | N/A | ++ | N/R | + | - | - | + | - |
| Hickson et al 2015 | N/R | + | - | N/A | + | N/A | ++ | ++ | + | ++ | **−** | N/A | ++ | N/R | ++ | ++ | + | ++ | + |
| Hilliam et al 2011 | - | - | - | N/A | + | - | N/R | ++ | + | ++ | **−** | N/A | ++ | N/R | - | - | - | - | + |
| Hirshfield et al 2012 | N/R | + | - | ++ | + | ++ | ++ | + | + | + | **−** | ++ | ++ | ++ | ++ | ++ | + | + | + |
| James, 2016 | Not assessed- abstract only | | | | | | | | | | | | | | | | | | |
| McOwan A et al 2002 | ++ | + | ++ | ++ | + | + | N/R | ++ | - | + | **−** | N/A | N/A | N/R | - | + | + | + | + |
| Pedrana et al 2012 | + | + | + | N/A | + | N/A | ++ | + | + | + | - | N/A | ++ | N/R | ++ | ++ | + | ++ | + |
| Prati et al 2016 | - | ++ | ++ | N/A | + | N/A | ++ | + | + | ++ | - | ++ | + | N/R | + | ++ |  | + | ++ |
| Solorio et al 2016 | + | + | + | N/A | ++ | N/A | + | + | + | ++ | - | N/A | + | N/R | - | ++ | + | + | - |
| Tang et al 2016 | N/R | + | ++ | ++ | - | N/R | - | + | + | + | - | ++ | + | N/R | - | + | + | + | + |
| Thackeray et al 2011 | Case Study- not quality assessed | | | | | | | | | | | | | | | | | | |
| West et al 2015 | Not assessed- abstract only | | | | | | | | | | | | | | | | | | |
| Wilkinson et al 2016 | - | ++ |  | N/A | + | N/A | ++ | + | + | ++ | - | N/A | + | N/R | ++ | ++ | + | ++ | + |
